# Estimation of Selection Intensity Against Dark Color Forms of the Spittlebug *Philaenus spumarius* (L.) in a Warming Climate

**DOI:** 10.3390/insects17030263

**Published:** 2026-03-01

**Authors:** Vinton Thompson

**Affiliations:** Division of Invertebrate Zoology, American Museum of Natural History, 200 Central Park West, New York, NY 10024, USA; vthompson@amnh.org

**Keywords:** climate change, color polymorphism, melanism, *Philaenus spumarius*, selection coefficient, selective sweep

## Abstract

Climate warming puts new selective pressures on natural populations, but there are few quantitative measurements of selection in natural populations over protracted time periods. Observations made at the beginning and end ofa 47-year cumulative increase of 2.7 °C in the mean September temperature in Northern Minnesota, USA, permit quantitative estimation of selection against a suite of alleles at a single locus determining the expressionof dark color forms in populations of the meadow spittlebug, *Philaenus spumarius* (L.) (Hemiptera: Cercopoidea: Aphrophoridae). Alternative methods of estimation of the selection coefficient *s*, a measure of the intensity of selection, produce values of *s* = 0.0125 and 0.0218, respectively, corresponding to a disadvantage of about one to two percent per year or, since *P. spumarius* is univoltine, per round of selection. The existence of a locus under selection presents an opportunity for molecular localization and characterization of the genetic locus determining color form. *Philaenus spumarius* is of particular interest in Europe, as it is the major local vector of the bacterial plant pathogen *Xylella fastidiosa*.

## Simple Summary

When the climate warms, natural selection may work against previously favored insect characteristics. Spittlebugs are sapsucking insects that live on plants. In the meadow spittlebug, the meadow spittlebug, a species found in Eurasia, North America and New Zealand, individuals exhibit one of several distinct color patterns determined by alternative forms of a single gene. Some have a dark coloration that absorbs more sunlight, giving them an advantage in cold environments, where dark forms tend to be more common. Over a 47-year interval in Northern Minnesota, USA, the mean temperature increased by 2.7 °C, while the proportion of genetically determined dark-color forms dropped by about a third, which is believed to be the result of selection against dark forms. This analysis estimates the intensity of natural selection against the dark forms, providing a quantitative measure of long-term biological change attributable to climate warming. The biology of the meadow spittlebug is of particular agricultural interest because it can infect plants, such as olive trees and grapevines, with a bacterium that causes serious diseases.

## 1. Introduction

By altering natural habitats, climate change puts new selection pressures on insect populations [1,2,3,4]. Warming environmental temperatures are the simplest, most direct effect. In Northern Minnesota, between 1974 and 2021, populations of the meadow spittlebug, *Philaenus spumarius* (L.) (Hemiptera: Cercopoidea: Aphrophoridae), exhibited a steep drop in the frequency of dark color forms concomitant with a 2.7 °C rise in the mean September temperature [5]. The decline in the frequency of dark color forms can most simply be interpreted as a response to elevation in local temperature, a manifestation of more widespread global warming. The aim of the present work is to provide a quantitative estimate of the magnitude of selection against dark color forms in the context of climate change.

Though there are many reported cases of selection in nature [6,7,8], instances in which selection has been measured for phenotypes determined by Mendelian loci are relatively few. In a thorough literature review, Thurman and Barrett [9] report 38 such studies, with 336 estimates of selection. Most involve color polymorphism in a handful of species, including, prominently and repeatedly, the paradigmatic peppered moth, *Biston Betularia* (L.), but also the moth *Callimorpha dominula* (L.) and species of *Cepaea* (snails) and *Adalia* (ladybird beetles). The study of color polymorphisms is an important source of insights into the workings of evolution and natural selection [10].

**Figure 1:**
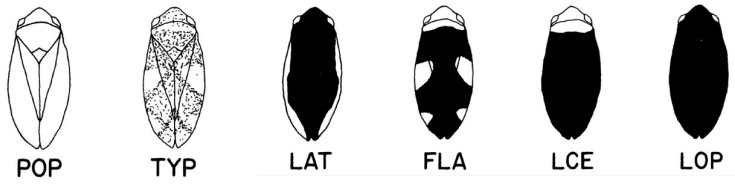
Stylized drawings of color forms present at frequencies greater than 1% in Northern Minnesota populations of the meadow spittlebug, *Philaenus spumarius*. The three-letter standardized abbreviations stand for the color forms populi (POP), typicus (TYP), lateralis (LAT), flavicollis (FLA), leucocephalus (LCE), and leucophthalmus (LOP). POP and TYP are non-melanic forms. LAT, FLA, LCE, and LOP are melanic forms. Modified from ref. [13]. In the living animals, the colors are a mix of cream, tan, brown, and black (see Figure 2).

**Figure 2:**
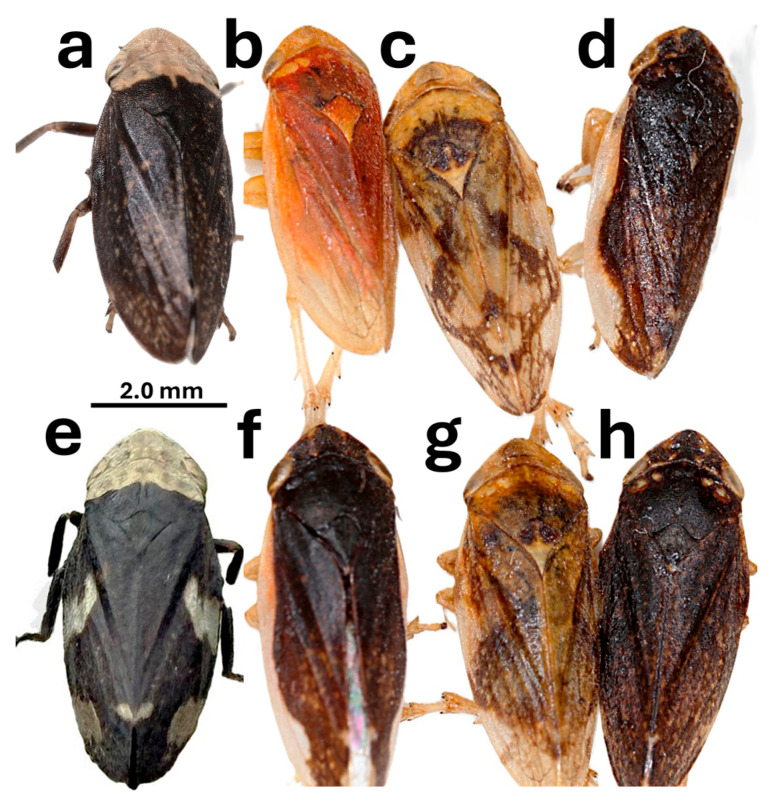
A montage of *Philaenus spumarius* specimens with different colors, giving a sense of the great variability of the species and the contrast between melanic and non-melanic forms. (**a**) LCE, (**b**) POP, (**c**) TYP, (**d**) LAT, (**e**) FLA, (**f**) LAT, (**g**) TYP, and (**h**) LOP. Individuals range from about 4.5 to 6.0 mm in length. Image (**a**) and collective image (**b**–**d**,**f**–**h**): © Gernot Kunz; image (**e**): © Paula Reed. All images were reproduced with permission.

*Philaenus spumarius* is highly polymorphic in terms of dorsal coloration. It exhibits an array of color forms (Figure 1 and Figure 2) controlled by multiple alleles at a single Mendelian locus [11,12]. While this polymorphism is probably influenced by a variety of selective factors, including crypsis and warning coloration [13], there is a strong association between climate and the frequency of several color forms; the colder the climate, the greater the frequency of dark forms [14,15]. Dark coloration is hypothesized to be an adaptation to cold temperature, permitting darker individuals to warm up faster and maintain higher body temperatures at critical times of the day and year [16]. This gives animals with darker color forms a relative selective advantage in cold climates, a phenomenon termed thermal melanism [17]. Females retain eggs until oviposition in the fall, making fall temperatures particularly important to reproductive success [5,13], hence the choice of September temperature as the measure of climate change.

Between 1974 and 2021, the combined frequency of melanic forms in the Minnesota population declined by about one third, falling from 22.1% to 14.2% in females and 12.7% to 7.3% in males. This highly significant drop in frequency (*p* = 0.001, Fisher’s exact test) [5] offers an unusual opportunity to estimate the magnitude of selection underlying the decrease. *Philaenus spumarius* is of particular interest because it is the primary European vector of the bacterial plant pathogen *Xylella fastidiosa*, which has badly damaged olive trees in Italy and olives, almonds, and grapes in Spain [13,18].

## 2. Materials and Methods

*Philaenus spumarius* polymorphism is complex. For the purposes of analysis, the six Northern Minnesota color forms (Figure 1) are consolidated into two categories: melanic and non-melanic. The two non-melanic phenotypes, POP and TYP (Figure 1), are determined by a single recessive allele and manifest as one or the other phenotype in different individuals. They are discrete in males but intergrade in females. The four melanic forms, LAT, FLA, LCE and LOP (Figure 1), are determined by four alleles, respectively, each dominant over the non-melanic allele and codominant with respect to one another [11]. The alleles determining the LAT, FLA and LOP phenotypes are not expressed in males. Consolidation of the color forms into two categories permits modeling of the locus as a simple two-allele system, with a collective dominant melanic allele and a recessive non-melanic allele. All the data underlying this analysis, including collection sites, phenotypic frequencies, and sample sizes, were taken from ref. [5].

Haldane [19] introduced the concept of the selection coefficient (*s*) as a quantitative measure of selection. He defined it as the difference in relative fitness between the fittest genotype and an alternative genotype under selection. By letting *s* represent the selective disadvantage of the melanic phenotypes and following Clarke and Murray’s approach [20], the genotypes and relative fitnesses can be expressed as follows:

Genotype  TT     Tt   tt 

Fitness    1−s   1−s   1

where T represents the melanic allele (in the present case, a collection of equivalent alleles), t represents the non-melanic allele, and *s* is the selection coefficient.

The single-generation gene frequency change will be

Δq = *s*q^2^(1 − q)/1 − *s*(1 − q^2^) (1)

where q is the frequency of the t allele.

Integration permits expression of the selection coefficient in terms of the initial and final values of q over n generations of constant selection:

*s* = [log_e_[q_n_(1 − q_0_)/q_0_(1 − q_n_)] − 1/q_n_ + 1/q_0_]/[n + log_e_(q_n_/q_0_) − 1/q_n_ + 1/q_0_] (2)

where q_0_ is the frequency of t at the start of selection, and q_n_ is the frequency at the end.

## 3. Results

Gavin the 1974 and 2021 Minnesota sample sizes of 389 and 608 females, respectively, estimated frequencies of t can be calculated as the square root of the frequency of the combined recessive non-melanic phenotypes. This estimate must be based on female counts because three of the four melanic alleles are not expressed in males. For 1974, the estimated q = 0.883 ± 0.016 (SE). In 2021, after 47 generations had passed, the estimated q = 0.927 ± 0.011 (SE). These estimates assume that the populations in question are in Hardy–Weinberg equilibrium and interlinked sufficiently to form a large panmictic population, assumptions likely met in close approximation because *P. spumarius* is an abundant, weedy species, forming large local populations linked through disturbed roadside habitats radiating throughout the area. Insertion of the initial and final q values into Equation (2) leads to the estimated value *s* = 0.0218 ± 0.0011(SE). The standard error was calculated using a complex equation for the variance of *s*, V(s), given by Clarke and Murray [20]. V(s) = 0.000627, leading to a standard deviation of V(s)^−2^ = 0.0125 and a standard error of about 0.0011.

The Clarke–Murray integral equation gives an approximation of *s* for recurring selection over multiple generations. Cook [21] recommends an alternative, more direct calculation of *s* based on iterative repeats of Equation (1). In this method, q_0_ is used to calculate a one-generation change in *q*. This is applied to generate a new value of *q* after one generation of selection, which in turn is used to generate the next-generation value of *q*, and so on (in this case, for 47 generations). Since the beginning (q_0_ = 0.883) and end (q_n_ = 0.927) frequencies are known, substitution of test values of *s* in Equation (1) permits iteration to a value of *s* that produces the observed outcome. Using this method, a set of recursion equations set up in Excel and reiterating Equation (1) forty-seven times led, after several trials, to an estimated *s* = 0.0125, which is a bit lower than the value produced by the Clarke–Murray method but of the same order of magnitude. There does not seem to be an established way to apply error limits to this calculation. Cook [21] refers readers back to the integral method for error estimates.

## 4. Discussion

### 4.1. Comparison to Other Estimations of Selection Coefficients

The estimated selection coefficient of 0.0125–0.0218 corresponds to a one-to-two-percent selective disadvantage for the combined melanic alleles. This falls at the low but well-populated end of observed exponential distributions of the range of *s* values for a wide variety of genes in natural populations, including both Mendelian and molecular loci [9]. It is consistent with the inference that slowly increasing temperatures have selected against dark color forms, ultimately manifesting in the highly significant net drop in the frequency of melanic forms between 1974 and 2021 [5].

How does this estimate compare to other measurements of selection intensity on traits determined by Mendelian phenotypes? Haldane estimates that *s* = 0.332 for selection favoring the melanic color form in *Biston betularia* [19]; this is a much stronger degree of selection than that reported here. For a subsequent period of decline in melanism in the same species, with selection against the melanic form, Cook [22] estimated values of *s* ranging from 0.018 to 0.208 for 26 locations in the UK, with a mean value of 0.113. For the snail *Cepaea nemoralis* (L.), Clarke and Murray [20] estimate that *s* = 0.061 for selection against a dominant allele for dark-brown coloration and 0.052 for selection favoring a single-banded shell allele in the same species. Stine and Smith [23] estimate that *s* = 0.34 for selection against the dominant allele causing Huntington disease and about 0.07 for selection against alleles causing porphyria variegata and lipoid proteinosis, respectively, among South African Afrikaners. Using an alternative approach based on analysis of computer-generated frequency surfaces for change over multiple generations, Cook et al. [24] estimated that *s* = 0.12 for selection against the melanic form in *Biston betularia*, and Brakefield and Lees [25] estimated that *s* = about 0.10 for selection against melanic forms of the ladybird beetle *Adalia bipunctata* (L.).

### 4.2. Assumptions Underlying the Selection Estimates

These estimates rest on a number of assumptions. They assume there are discrete generations, an assumption met by *P. spumarius*, which is univoltine, with a single generation per year. In contrast, *Biston betularia*, *Cepaea* species, and *Adalia* species have multiple, overlapping generations per year. This necessitates estimation of generations elapsed, generating an extra source of uncertainty. The estimates also assume that selection is constant during the time interval measured. The Minnesota localities hosting *P. spumarius* warmed unevenly over the 47-year period studied, with intermediate dips and spikes in temperature [5]. However, the long-term averaging implicit in the assumption of constant selection generally reflects biological reality [9] and is unlikely to seriously compromise the general results.

The present study also tacitly assumes that the genetics of color-form determination are the same in both the North American populations studied and the Finnish and British populations for which breeding tests originally determined patterns of inheritance. This is almost certainly true because there is strong evidence that North American populations of *P. spumarius* originated from relatively recent human-mediated introductions from Europe [26,27].

Finally, while it is suggested that the association between environmental warming and the decline in the frequency of dark color forms is causal, causality is inferred, not demonstrated. The decline could be due to other, unknown causes, which could be coincidentally correlated with temperature increase. Many other factors probably influence the frequency of color forms [13], including frequency-dependent selection for rarity [28], a form of balancing selection as opposed to the directional selection inferred here. It is impossible to exclude the possibility that concomitant environmental changes, related or unrelated to climate warming, caused the observed decline in melanic forms. Because the populations are large, the change is unlikely to be the result of random processes like genetic drift. Temporal stability in color-form frequencies is the general rule for *P. spumarius* [12]. Departures from stability invite explanation.

### 4.3. Molecular Estimates of Selection Intensity and Prospects for Using Selective Sweep Analysis to Identify the P. spumarius Color Form Locus

Recently, new molecular approaches have revolutionized the study of selection in natural populations [9,29]. Whole-genome data permit simultaneous estimation of selection coefficients for hundreds or thousands of loci, dwarfing the scale of classical investigations based on Mendelian phenotypes. For example, a single study [30] produced 2793 selection coefficients [9], an approach likely to be enhanced by the application of artificial intelligence [31]. Campagna et al. [32] have demonstrated the applicability of these tools to the study of the evolution of melanism in the Chestnut-bellied monarch (*Monarcha castaneiventris* Verreaux), a Solomon Islands bird with multiple subspecies color forms. They estimated selection coefficients favoring alleles determining melanistic forms of approximately *s* = 0.01–0.02 over intervals of 500–2200 generations, values of the same magnitude as those reported here but hypothesized to have taken effect over considerably longer time periods.

Despite some effort, the nature and location of the Mendelian locus determining the color forms of *P. spumarius* are still unknown [33]. New molecular techniques that detect loci under selection based on identification of selective sweeps may offer an opportunity to pinpoint this gene [31,34]. While the Minnesota populations are a possible target for such analysis, *P. spumarius* populations in the Cynon Valley in Wales, the UK, appear to offer the most promising opportunity. This locality has the highest frequency of melanic forms ever recorded [11], but the frequency has dropped from over 90% to about 50% over 33 generations [35]), indicative of strong selection. These populations appear to be ripe for a genetic sweep analysis, particularly for “soft sweeps,” which result when alleles already present in populations in considerable frequency come under selection [34].

### 4.4. Selection Against P. spumarius Melanic Color Forms Is Just One Instance of Climate-Driven Change in Insect Coloration

*Philaenus spumarius* is not the only polymorphic insect showing evidence of long-term decline in darker color forms as a result of climate warming. The same phenomenon has been documented in the leaf beetle *Chrysomela lapponica* L. (Coleoptera: Chrysomelidae) over a 26-year period in the Kola Peninsula of Russia [36,37]. Additionally, Clusella-Trullas and Nielsen [2] reviewed many other cases of shifts in dark coloration attributed to climate change in a wide variety of other insects, including cases in which melanization is a continuous characteristic within species [38] and cases in which the predominance of melanized species changes in multispecies assemblages [1]. Among the many insects studied to date, *P. spumarius* stands out for its widespread occurrence, abundance, and importance as the major vector of *Xylella fastidiosa* in Europe [13,18].

## Data Availability

The original contributions presented in this study are included in the article. Further inquiries can be directed to the author.
